# Assessment of Quality of Life Among Type 2 Diabetes Mellitus Patients in an Urban Health Center of Tiruvallur District, Tamil Nadu

**DOI:** 10.7759/cureus.63320

**Published:** 2024-06-27

**Authors:** Rex Vijay V, Praveen Kumar I, Buvnesh Kumar M, J Sagetha

**Affiliations:** 1 Department of Community Medicine, Saveetha Medical College and Hospital, Saveetha Institute of Medical and Technical Sciences (SIMATS) Saveetha University, Chennai, IND

**Keywords:** health center, quality of life, who-bref, diabetic complications, lifestyle modifications

## Abstract

Introduction

Diabetes mellitus (DM) is one of the leading causes of death and disability, in addition to its rapidly increasing prevalence in developing countries. The key element of managing diabetes is quality of life (QoL). It is a factor that is positively related to treatment adherence, and QoL motivates the patient to manage their disease and achieve health and happiness in the long term. We undertook this study in a district of South India among patients with type 2 diabetes mellitus (T2DM) to assess their QoL.

Methodology

A cross-sectional study was conducted among T2DM patients registered in a noncommunicable disease (NCD) clinic in an urban health center in Tiruvallur District, Tamil Nadu. Participants were selected using a systematic random sampling method from the NCD clinic register. Sociodemographic details of the participants were collected using a pretested, semistructured questionnaire, following which the World Health Organization Quality of Life-Brief Version Scale (WHOQOL-BBREF) questionnaire was used to assess the QoL. Data was entered in MS Excel (Microsoft Corporation, Redmond, Washington, United States) and analyzed using IBM SPSS Statistics for Windows, Version 25.0 (Released 2017; IBM Corp., Armonk, New York, United States).

Results

The mean age of the study participants was 53.5 ± 9.2 years. Females constituted 63.81% of the study population compared to males (36.19%). Domain-wise, 55.83% had good physical QoL, 49.1% had good psychological QoL, 49.69% had good social QoL, and 57.1% had good environmental QoL. Overall, 52.8% had a good QoL across all the domains.

Conclusion

The current study found that patients with diabetes had a good QoL with the exception in educational category. However, it is essential to create an awareness among the diabetic patients about the QoL and also the measures that they can practice to improve their QoL, which should be emphasized through health education, information education communication (IEC), and counselling in NCD clinics.

## Introduction

Diabetes mellitus (DM) is a syndrome of multiple etiologies characterized by chronic hyperglycemia with disturbances of carbohydrate, fat, and protein metabolisms resulting from defects in insulin secretion, insulin action, or both [1]. A DM patient is a person who is already diagnosed with DM. A person is considered to be diabetic when his/her random blood sugar levels are ≥ 126 mg/ dl and/or using insulin or an oral hypoglycemic agent [1]. Diabetes is a chronic disease that occurs either when the pancreas does not produce enough insulin or when the body cannot effectively use the insulin it produces [2]. Type 2 diabetes mellitus (T2DM) affects how your body uses sugar (glucose) for energy and stops the body from using insulin properly, which can lead to high levels of blood sugar if not treated [2]. T2DM can cause severe harm to the body, particularly nerves and blood vessels [2].

Diabetes mortality rate increased by 3% in each age group between 2000 and 2019 [2]. Compared to high-income countries, the prevalence has been rising rapidly in low- and middle-income countries [3]. Globally, 643 million people will have diabetes by 2030, and by 2045, that number will rise to 783 million. India has the second-highest rate of people with diabetes worldwide. In 2019, the number of disability-adjusted life years (DALYs) due to T2DM worldwide was 66.3 million, with an age-standardized rate of 801.5 DALYs per 100,000 population, an increase of 27.6% since 1990 [4]. According to the National Family Health Survey-5 (NFHS-5), 7.2% of men and 6.3% of women were found to have very high blood sugar levels, with the prevalence being high in urban areas. This indicator has been added to the NFHS-5. A total of 13.5% and 15.6% of the women and men, respectively, are on regular medications. Tamil Nadu had a relatively higher prevalence of 11.1% and 11.9% of women and men having very high blood sugar levels, respectively, with urban and rural areas showing no difference in the rates. A total of 20.7% of the women and 22.1% of the men were on regular medications for T2DM [5].

Every fifth diabetic in the world is an Indian, with rising trends caused by aging, obesity, physical inactivity, genetic predisposition, rural-to-urban migration, and family history [6]. Diabetes is the leading cause of strokes, heart attacks, kidney failure, blindness, and amputation of lower limbs. Therefore, assessing the quality of life (QoL) of patients is important because each individual has an individualized perception of their physical, emotional, and social well-being, which includes a cognitive element of satisfaction as well as an emotional component of happiness [7].

The World Health Organization (WHO) has provided a comprehensive definition for QOL: “individuals’ perception of their position in life in the context of the culture and value systems in which they live and in relation to their goals, expectations, standards, and concerns” (The WHOQOL Group, 1995). More than half of the people are unaware that they have diabetes, which can lead to health problems if not detected and treated in a timely manner. Various factors associated with both short-term and long-term diabetes management influence their QoL negatively or positively [6,8]. The primary elements influencing a patient's QoL are the disease's longer duration of illness and its microvascular and macrovascular consequences [9]. A patient's commitment to managing his illness may also be significantly impacted by a deteriorating QoL and depression [6]. Physically, it causes discomfort, incapacity, and reduced mobility due to neuropathy, retinopathy, nephropathy, cardiovascular disease, and foot problems. Diabetes generates emotional stress, anxiety, and sadness since it demands continual monitoring and concerns about complications [10,11]. It might lead to social isolation and make participation in activities more challenging. The cost of diabetic control, as well as issues with self-management, sleep disruptions, has effects on productivity at work, all adding to stress.

T2DM patients suffer both psychologically and physically; hence, the QoL for those patients suffering from the illness should be a top priority in disease management. QoL should be taken into consideration for people suffering from complications due to diabetes, such as diabetic retinopathy, nephropathy, and neuropathy, since these patients will be undergoing more psychological changes and stress [12]. QoL is a central issue for patients, caretakers, and health care workers. It is essential and the need of the hour to create an awareness among the patients about their QoL and the measures that they can practice for improving their QoL, which should be emphasized through health education, information education and communication (IEC), and counselling in noncommunicable disease (NCD) clinics [13]. The objective of the study is to assess the association of sociodemographic factors among the T2DM patients with the QoL. We aimed to study QoL among diabetic patients visiting NCD clinics in an urban health center.

## Materials and methods

A cross-sectional study was conducted in an urban health training center of a tertiary care hospital in Tiruvallur District, Tamil Nadu. The study was conducted from January 2023 to April 2023, for four months. All T2DM patients who attended the NCD clinic and were willing to participate in the study by providing their valid consent were included as the study subjects. Study participants were selected using systematic random sampling.

Considering the prevalence of diabetes from the study conducted by Sun et al. [14] as 10.8% with an alpha error of 0.05, the power of the study at 80% with a 95% confidence level, and a nonresponse rate of 10%, the minimum sample size required for the study was calculated as 163 by using the OpenEpi (v 3.01) sample size calculator. Based on the anticipated prevalence of diabetes as 10.8% with an alpha error of 0.05, 80% power, and 95% confidence interval, the minimum sample size required for the study was 148 by using the formula. Adding a 10% nonresponse rate to this, the minimum sample size was calculated as 163, which was rechecked manually using the formula for a cross-sectional study: n = (Z_1 -α/2_)2 * P*Q/d2, where P = 10.8, making it^ ^n = 3.84*10.8*89.2/(5)2, and finally n = 148, wherein Z_1 -α/2 _is the standard normal variate (at 5% type 1 error (p < 0.05), making it 1.96; P is the expected prevalence from previous studies; Q = 1 - P; d is the allowable error; and n is the sample size.

Systematic random sampling was employed to select the study participants. Initially, the total number of patients with T2DM during the study period was calculated from the NCD register of the urban health center. Then, the sampling interval (k) was determined by dividing the total number of eligible individuals by the sample size to be drawn. The first participant was randomly selected between one and k. Subsequently, the k value was added until the proposed sample size was reached. The total number of patients registered in the NCD clinic was 452, and the sampling interval obtained was k = 3. Every third patient who came for consultation during the data collection period was recruited for the study. If a patient was not willing to participate, the next patient was chosen, and baseline sociodemographic data was collected using a pretested, semistructured questionnaire and interview method. Valid consent was obtained, and each participant received a description of the study in their own language. We recruited 163 participants for our study, as we received extra responses and have included them in our analysis.

The World Health Organization Quality of Life-Brief Version Scale (WHOQOL-BREF) questionnaire [15] was used to assess the QoL, which consists of four domains: physical health, psychological, social relationships, and environmental. There were also two items that were examined separately: question 1 about an individual's overall perception of QoL and question 2 about an individual's overall perception of his or her health. Domain scores were scaled in a positive direction (i.e., higher scores denote a higher QoL). The domain score was determined by taking the average of the items within each domain. After multiplying the mean scores by four, domain scores were made comparable to those used in the WHOQOL-100. The resulting 0-100 scale was then converted using the previously mentioned formula.

The data was collected using the interview method, entered in MS Excel (Microsoft Corporation, Redmond, Washington, United States) and analyzed using IBM SPSS Statistics for Windows, Version 25 (Released 2017; IBM Corp., Armonk, New York, United States). Descriptive statistics were used to analyze the baseline details, and a chi-square test was used to find the association between QoL and sociodemographic variables. The study was conducted after obtaining ethical approval from the Institutional Ethical Committee. Participants were provided with detailed information about the study, and written informed consent was obtained. Participants identity was always kept confidential, and the data was used only for research purposes.

## Results

The mean age of the study participants was 53.5 ± 9.2 years. Table 1 shows that females constituted 63.81% of the study population compared to males, who constituted 36.19%. The participants had varying levels of educational qualifications, with the highest percentage having secondary education (33.12%), followed by undergraduate (27.63%), primary (27.6%), and the lowest percentage being illiterate (11.65%). A total of 11% of the study population were unmarried, and 67.5% of the study population were married. According to the modified Brahm Govind (BG) Prasad classification for October 2023 [16], 37.4% of the participants belonged to the middle class, 35% belonged to the lower middle class, and 13.5% belonged to the lower class.

**Table 1:** Sociodemographic details of the study participants *Socioeconomic status according to the modified Brahm Govind (BG) Prasad scale, October 2023 T2DM: Diabetes mellitus

Variable	Category	Frequency (N = 163)	Percentage (%)
Gender	Male	59	36.19
	Female	104	63.81
Educational qualification	Illiterate	19	11.65
	Primary	45	27.6
	Secondary	54	33.12
	Undergraduate	45	27.63
Marital status	Single	18	11
	Married	110	67.5
	Divorced	17	10.4
	Widowed	18	11.1
H/o comorbidity other than T2DM in the last year	Yes	104	63.8
	No	59	36.2
Socioeconomic status^*^	Class 1	11	6.7
	Class 2	12	7.4
	Class 3	61	37.4
	Class 4	57	35
	Class 5	22	13.5

The scores obtained by the participants were converted into transformed scores, and the mean score for each domain was calculated. The mean (standard deviation) of the physical health domain was found among the participants using the WHO BREF questionnaire was found to be 19.63 (6.57). The mean (SD) of the other three domains, namely, mental health, social relationships, and environmental domain, were found to be 16.76 (5.62), 7.91 (3.43), and 20.64 (5.91), respectively. The mean total score of the QOL scale was 16.23, and the standard deviation was 5.38.

The QoL scores were further converted into a categorical variable by dividing the group into those who got a score above the mean and those below the mean. They were labelled as having good and poor QoL. The number of participants who got a good QoL (>50%) in the physical health domain was 55.83%, and 44.17% got poor QoL (<50%). In the mental health domain, the number of participants who got good QoL was 49.1%, and the number of participants who got poor QoL was 50.9%. In the social relationship domain, 49.69% of participants got a good QoL, and 50.31% got a poor QoL. In the environmental domain, 57.1% of participants got good QoL, and 42.9% got poor QoL.

Table 2 shows distribution of body mass index (BMI) and duration of T2DM among the study participants. Among the study participants, 33.74% and 38.12% belong to overweight and obese category, respectively. About 53.8% of the study participants are having T2DM in less than five years, and about 46.62% of the study participants have T2DM >/= 5 years.

**Table 2:** Distribution body mass index (BMI) and duration of T2DM among the study participants (N = 163) T2DM: Type 2 diabetes mellitus

Variable	Category	Frequency (%) (N = 163)
BMI	Underweight	8 (4.91)
	Normal	38 (23.23)
	Overweight	55 (33.74)
	Obese	62 (38.12)
Duration of T2DM	< 5 years	87 (53.38)
	>/= 5 years	76 (46.62)

Table 3 shows the association of sociodemographic factors with the physical health domain. A total of 52.5% of males and 57.7% of females had good physical health and QoL. A p-value of 0.525 showed that there was no significant association between gender, physical health, and QoL. Among different educational qualifications, 100% of illiterates had a good physical health QoL, whereas the percentages for elementary, secondary, and undergraduate qualifications were 55.6%, 42.6%, and 53.3%, respectively. The chi-square value of 18.984 with a p-value of <0.01 suggested a significant association between educational qualification and physical health QoL. The data showed no significant difference in the physical health QoL based on the marital status or the current illness of patients. The individuals in Class 1 and Class 5 socioeconomic status had higher percentages of good QoL compared to other classes. The chi-square value of 3.263 with a p-value of 0.515 indicated no significant association between socioeconomic status and physical health QoL. In summary, educational qualification had a significant association with physical health QoL, while gender, marital status, comorbidity status, and socioeconomic status did not show significant associations with physical health outcomes in the study population.

**Table 3:** Association of sociodemographic factors with the physical health domain *p-value < 0.05 is significant; *socioeconomic status according to the modified Brahm Govind (BG) Prasad scale, October 2023 T2DM: Type 2 diabetes mellitus; QoL: quality of life

Variable	Category	QoL		Chi-square	p-value
		Good N = 91 (%)	Poor N= 72 (%)		
Gender	Male	31 (52.5)	28 (47.5)	0.405	0.525
	Female	60 (57.7)	44 (42.3)		
Educational qualification	Illiterate	13 (68.42)	6 (31.58)	7.984	*0.04
	Primary	25 (55.6)	20 (44.4)		
	Secondary	23 (42.6)	31 (57.4)		
	Undergraduate	24 (53.3)	21 (46.7)		
Marital status	Single	9 (50)	9 (50)	0.801	0.849
	Married	64 (58.2)	46 (41.8)		
	Divorced	9 (52.9)	8 (47.1)		
	Widowed	9 (50)	9 (50)		
H/o comorbidity other than T2DM in the last year	Yes	59 (56.7)	45 (43.3)	0.095	0.758
	No	32 (54.2)	27 (45.8)		
Socioeconomic status*	Class 1	5 (45.5)	6 (54.5)	3.263	0.515
	Class 2	6 (50)	6 (50)		
	Class 3	38 (62.3)	23 (37.7)		
	Class 4	28 (49.1)	29 (50.9)		
	Class 5	14 (63.6)	8 (36.4)		

Table 4 shows the association of sociodemographic factors with the mental health domain. From the table, only educational qualification had a significant association with mental health QoL, while gender, marital status, comorbidity status, and socioeconomic status did not show significant associations with mental health outcomes in the study population.

**Table 4:** Association of sociodemographic factors with the mental health domain *p-value < 0.05 is significant; *socioeconomic status according to the modified Brahm Govind (BG) Prasad scale, October 2023 T2DM: Type 2 diabetes mellitus; QoL: quality of life

Variable	Category	QoL		Chi-square	p-value
		Good N = 80 (%)	Poor N = 83 (%)		
Gender	Male	28 (47.5)	31 (52.5)	0.097	0.755
	Female	52 (50)	52 (50)		
Educational qualification	Illiterate	16 (84.21)	3 (15.79)	8.094	*0.007
	Primary	21 (46.7)	24 (53.3)		
	Secondary	19 (35.2)	35 (64.8)		
	Undergraduate	21 (46.7)	24 (53.3)		
Marital status	Single	7 (38.9)	11 (61.1)	1.261	0.739
	Married	57 (51.8)	53 (48.2)		
	Divorced	8 (47.1)	9 (52.9)		
	Widowed	8 (44.4)	10 (55.6)		
H/o comorbidity other than T2DM in the last year	Yes/No	53 (51)	51 (49)	0.407	0.523
		27 (45.8)	32 (54.2)		
Socio economic status*	Class 1	5 (45.5)	6 (54.5)	3.215	0.523
	Class 2	4 (33.3)	8 (66.7)		
	Class 3	34 (55.7)	27 (44.3)		
	Class 4	25 (43.9)	32 (56.1)		
	Class 5	12 (54.5)	10 (45.5)		

Table 5 indicates that educational qualification had a significant association with social health QoL, while gender, marital status, comorbidity status, and socioeconomic status did not show significant associations with social health outcomes in the study population.

**Table 5:** Association of sociodemographic factors with the social domain *p-value < 0.05 is significant; *socioeconomic status according to the modified Brahm Govind (BG) Prasad scale, October 2023 T2DM: Type 2 diabetes mellitus; QoL: quality of life

Variable	Category	QoL		Chi-square	p-value
		Good N = 81 (%)	Poor N = 82 (%)		
Gender	Male	28 (47.5)	31 (52.5)	0.185	0.667
	Female	53 (51)	51 (49)		
Educational qualification	Illiterate	17 (89.47)	2 (10.53)	9.958	*0.005
	Primary	21 (46.7)	24 (53.3)		
	Secondary	19 (35.2)	35 (64.8)		
	Undergraduate	22 (48.9)	23 (51.1)		
Marital status	Single	8 (44.4)	10 (55.6)	0.643	0.887
	Married	57 (51.8)	53 (48.2)		
	Divorced	8 (47.1)	9 (52.9)		
	Widowed	8 (44.4)	10 (55.6)		
H/o comorbidity other than T2DM in the last year	Yes	54 (51.9)	50 (48.1)	0.571	0.451
	No	27 (45.8)	32 (54.2)		
Socioeconomic status*	Class 1	5 (45.5)	6 (54.5)	2.379	0.666
	Class 2	4 (33.3)	8 (66.7)		
	Class 3	34 (55.7)	27 (44.3)		
	Class 4	27 (47.4)	30 (52.6)		
	Class 5	11 (50)	11 (50)		

Table 6 indicates that educational qualification had a significant association with environmental health QoL, while gender, marital status, comorbidity status, and socioeconomic status did not show significant associations with environmental health outcomes in the study population.

**Table 6:** Association of sociodemographic factors with the environmental domain *p-value < 0.05 is significant; *socioeconomic status according to the modified Brahm Govind (BG) Prasad scale, October 2023 T2DM: Type 2 diabetes mellitus; QoL: quality of life

Variable	Category	QoL		Chi-square	p-value
		Good N = 93 (%)	Poor N = 70 (%)		
Gender	Male	31 (52.5)	28 (47.5)	0.769	0.381
	Female	62 (59.6)	42 (40.4)		
Educational qualification	Illiterate	18 (94.7)	1 (5.3)	13.644	*0.003
	Primary	25 (55.6)	20 (44.4)		
	Secondary	25 (46.3)	29 (53.7)		
	Undergraduate	25 (55.6)	20 (44.4)		
Marital status	Single	9 (50)	9 (50)	1.238	0.744
	Married	66 (60)	44 (40)		
	Divorced	9 (52.9)	8 (47.1)		
	Widowed	9 (50)	9 (50)		
H/o comorbidity other than T2DM in the last year	Yes	61 (58.7)	43 (41.3)	0.301	0.584
	No	32 (54.2)	27 (45.8)		
Socioeconomic status*	Class 1	6 (54.5)	5 (45.5)	2.859	0.582
	Class 2	5 (41.7)	7 (58.3)		
	Class 3	39 (63.9)	22 (36.1)		
	Class 4	30 (52.6)	27 (47.4)		
	Class 5	13 (59.1)	9 (40.9)		

## Discussion

The mean QoL BREF instrument score, indicating the QoL of the participants, was 52.8. Domain-wise, 55.83% had good physical QoL, 49.1% had good psychological QoL, 49.69% had good social QoL, and 57.1% had good environmental QoL. Overall, 52.8% had a good QOL across all the domains. An association was seen between sociodemographic factors and physical health, mental health, social, and environmental domains individually using the Chi-square test. Unlike other studies, associations were seen between every four domains individually with sociodemographic factors in this study.

The estimated QoL in our study was higher than the estimates of a similar study done by Suryavanshi et al. [17], though the study instrument used was different from our study. In a similar study done by Manjunath et al. to estimate the QoL of a patient with T2DM domain-wise, 63% had good physical QoL, 69% had good psychological QoL, 27% had good social QoL, and 85% had good environmental QoL [18]. A total of 68% of the patients reported having a good QoL overall. Compared with a study done by Sharma et al. [19], it was reported that 26.25% of the respondents felt that their QoL was slightly affected, whereas 25.93% responded that their QoL was moderately affected.

Studies have previously indicated a linear relationship between education level and health-related quality of life (HRQoL). This might be a result of their better understanding of the illness process, how it affects them, and their greatest efforts to maintain lifelong diabetes treatment [20,21]. In contrast to this, our study found that illiterate participants have a good QoL on all domain scores. This may be due to the participants who studied up to higher education tend to adopt a sedentary lifestyle, which may cause a lack of physical activity leading to risk factors for T2DM. A study conducted by Barua et al. [21] identified marriage as a predictor of HRQoL, but we did not find any significant variations in QoL scores with marital status across age groups in any domain.

Participants in a prior study by Barua et al. [21] who had T2DM for less than five years had a higher HRQoL than those who had the disease for more than five years [21]. This negative effect on HRQoL was also noted in earlier research [20]. Again, the results of this previous study regarding the effect of comorbidities on HRQoL are supported by two additional Asian studies [21-25]. However, we did not find any significant variations in QoL scores with the history of comorbidity status of study participants across all domains. In this regard, the review by Rubin and Peyrot indicates that the relationship between HRQoL and comorbidities is so strong and consistent that inconsistent findings about the relationship between other variables and QoL may be explained by the frequent omission of this factor as a potential confounding variable [21,26].

Our study did not reveal any significant differences in QoL scores across all domains, except for educational attainment, among the participants in all domains. This is in contrast to Gebremedhin et al. [27,28], who reported a significant relationship between age and all domains of QoL. Our study found an association of higher education on all domain scores, which is similar to the findings of Pandey et al. [28]. Despite having a good economic and educational background, diabetes-related complications can have a severe impact on patients' lives [28]. A meta-analysis of QoL studies among diabetic patients by Jing et al. revealed that hypertension and complications are significant negative determinants of QoL in individuals with T2DM individuals. However, in our study, we did not find any association between QoL and the participants’ comorbidity status of the study participants [28].

Proper management of a healthy lifestyle is crucial for successful treatment of diabetes. In certain cases, patients with T2DM may require medications such as insulin injections to regulate their blood sugar levels [29]. Additionally, medications may be necessary to control high blood pressure, and statins may reduce the risk of complications. Foot care, including the treatment of ulcers, kidney disease screening and treatment, and retinopathy screening via eye examination, may also be essential. The WHO is committed to promoting and supporting effective measures for the surveillance, prevention, and control of diabetes and its complications, particularly in low-income and middle-income countries. In April 2021, the WHO launched the Global Diabetes Compact, a global initiative aimed at improving diabetes prevention and care, with a particular focus on supporting low-income and middle-income countries [1]. In May 2021, the World Health Assembly passed a resolution to strengthen the prevention and control of diabetes [1]. In May 2022, the World Health Assembly endorsed the achievement of five global diabetes coverage and treatment targets by 2030. The Centers for Disease Control and Prevention (CDC) developed the National Diabetes Prevention Program (National DPP), a resource designed to implement evidence-based lifestyle change programs to prevent T2DM in communities.

The strength of this study stems from its randomized survey design, which guarantees dependable outcomes and uncovers the connection between DM and QoL [29]. The use of WHOQOL-BREF questionnaire as a study tool in assessing the QoL among T2DM patients may attribute to one of the strengths of this study. However, this study has some limitations. First, the generalizability of the study findings is limited because the study site was a tertiary care hospital with a small sample size and brief study duration. Second, the elderly participants might have taken longer to respond due to various associated factors, leading to a potential bias in the study results. Moreover, the original design did not include measurements of glucose values or hemoglobin A1C. Additionally, because the survey database eliminated private information, some subjects could not be sampled to measure their glucose values or hemoglobin A1C, which could result in an underestimation of the prevalence of undiagnosed diabetes in older individuals [30].

## Conclusions

With the increased prevalence of diabetes in India and worldwide, it is increasingly crucial to evaluate QoL as an outcome measure for long-term illnesses and management. In the present study, we found an association between educational qualification and QoL among the study participants. The association we got in educational qualification may imply the participant's incorporating the sedentary lifestyle due to the lack of regular physical activity. However, the associations observed are correlational and not necessarily indicative of direct effects. The limitation of the study is the cross-sectional design which is appropriate for identifying associations but not for establishing cause-effect relationships. Additionally, given the study's setting in a single urban health center, the generalizability of the findings to other settings or rural populations might be limited. The present study is one of the few studies conducted in India that examined the QoL of patients with diabetes. We recommend incorporating QoL assessments as part of diabetes treatment strategies. We can try prevention strategies through counseling about physical activity, and lifestyle modifications through IEC activities in preventing the development of T2DM. 

## Appendices

Data collection sheet

A.    Sociodemographic Characteristics

1.         Name of the study participant:

2.         Age:

3.         Gender: male/female/others

4.         Educational qualification: illiterate/primary and middle/secondary/graduate

5.         Marital status: single/married/divorced/widowed

6.         Socioeconomic status: Class 1/2/3/4/5

7.         History of diabetes mellitus in the last year: yes or no

8.         Monthly income of the family:
